# Suicide risk and crises suffered in the context of COVID-19 mediated by interpersonal family communication

**DOI:** 10.15446/rsap.V25n2.105378

**Published:** 2023-03-01

**Authors:** Miguel Garcés-Prettel, Elias Geney-Castro, Karol Gutiérrez-Ruiz, Yanin Santoya-Montes

**Affiliations:** 1 MG: Professional Educational Communication. Ph.D. Communication. Associate professor, Universidad Tecnológica de Bolívar. Cartagena de Indias, Colombia. mgarces@utb.edu.co Universidad Tecnológica de Bolívar Universidad Tecnológica de Bolívar Cartagena de Indias Colombia mgarces@utb.edu.co; 2 EG: Psychol. Ph.D. Psychology. Assistant professor, Universidad Tecnde Bolívar. Cartagena de Indias, Colombia. egeney@utb.edu.co Universidad Tecnde Bolívar Cartagena de Indias Colombia egeney@utb.edu.co; 3 KG: Psychol. Ph.D. Neuropsychology. Associate professor, Universidad Tecnológica de Bolívar. Cartagena de Indias, Colombia. kgutierrez@utb.edu.co Universidad Tecnológica de Bolívar Neuropsychology Universidad Tecnológica de Bolívar Cartagena de Indias Colombia kgutierrez@utb.edu.co; 4 YS: Psychol. M.Sc. Neuropsychology. Postgraduate professor, Universidad Tecnológica de Bolívar. Barranquilla, Colombia. yaninsantoya@gmail.com Universidad Tecnológica de Bolívar Neuropsychology Universidad Tecnológica de Bolívar Barranquilla Colombia

**Keywords:** Suicidal ideation, suicide, mental health, COVID-19, family relationships, communication *(source: MeSH, NLM)*, Ideación suicida, suicidio, expresión de preocupación, COVID-19, relaciones familiares, comunicación *(fuente: DeCS, BIEREME)*

## Abstract

**Objective:**

To analyze the relationship between suicidal ideation and the concerns or affectations perceived at the family, economic and health levels during the first year of the COVID-19 pandemic in Colombia, and the mediating role of family communication.

**Method:**

Participants were obtained through cluster sampling and quotas, resulting in 660 Colombians (Z=1.96; variance=0.25; error=3.9%), who answered a questionnaire consisting of three reliable scales between November 1 and 30, 2020, after providing informed consent. Binary logistic regression was used to evaluate the concerns or affectations that most influenced suicidal ideation during this period.

**Results:**

Thirty-five percent of the Colombian participants presented a high level of suicidal ideation. The concerns or affectations that most strongly explain this level come from the realm of health and intrafamily relationships. The effect of health concerns or affectations on suicidal ideation decreases by up to 32.4% when family communication is open. Likewise, when this type of positive communication is present, the effect of the concerns or affectations perceived in the family, such as breakdowns, distancing, and crisis of coexistence, on suicidal ideation decrease between 29.1% and 70.1%.

**Conclusion:**

Open family communication is a protective factor for mental health in crisis contexts because it can contribute to preventing suicide and alleviate to the concerns or affectations generated by COVID-19. Therefore, it is necessary to strengthen mental health programs through a communicative approach aimed at promoting assertive family dialog to achieve greater attention and openness to talk about concerns and affectations suffered.

## RESUMEN

**Objetivo:**

Analizar la relación entre la ideación suicida y las preocupaciones o afectaciones percibidas a nivel familiar, económico y de salud durante el primer año de la pandemia por COVID-19 en Colombia y el rol mediador de la comunicación familiar.

**Método:**

Los participantes se obtuvieron mediante muestreo por conglomerados y cuotas, de lo cual resultaron 660 colombianos (Z=1.96; varianza=0.25; error=3.9%) que respondieron a tres escalas confiables aplicadas en noviembre de 2020, previo consentimiento informado. Se utilizó regresión logística binaria para evaluar las preocupaciones o afectaciones que más influyeron en la ideación suicida en este período.

**Resultados:**

El 35% de los participantes presentó un nivel alto de ideación suicida. Las preocupaciones o afectaciones que más explican este nivel provienen del ámbito de la salud y las relaciones intrafamiliares. El efecto de las preocupaciones o afectaciones de salud sobre la ideación suicida disminuye hasta en un 32.4% cuando la comunicación familiar es abierta. Cuando está presente este tipo de comunicación positiva, el efecto de las preocupaciones o afectaciones percibidas en la familia, como rupturas, distanciamiento y crisis de convivencia, sobre la ideación suicida disminuye entre un 29.1% y un 70.1%.

**Conclusión:**

La comunicación familiar abierta es un factor protector para la salud mental en contextos de crisis sanitaria porque puede contribuir a prevenir el suicidio. Por ello, es necesario fortalecer los programas de salud mental a través de un enfoque comunicativo encaminado a promover el diálogo familiar asertivo para lograr una mayor atención y apertura para hablar de las preocupaciones y afectaciones sufridas.

The COVID-19 pandemic has impacted mental health worldwide 1. During this crisis, people have dealt with the fear of getting sick, misinformation, economic instability caused by work interruptions, the decrease or loss of income, restrictions on free movement and socialization and the presence of emotions such as anger, frustration, and distress 2. Added to this is the increase in stress and anxiety levels 3.

World mental health statistics estimate a prevalence of 15.97% for depression, 15.15% for anxiety, 23.87% for insomnia, 21.94% for posttraumatic stress disorder and 13.29% for psychological distress in the context of this pandemic 4. These data converge with the report of the World Health Organization (WHO), which shows that in the first year of the COVID-19 pandemic, anxiety and depression problems increased by 25% 5. This increase is associated with uncertainty and concern for personal health, economic concerns, and social distancing due to the pandemic 6,7.

It has been warned in this pandemic context that people with psychiatric disorders could experience a worsening of symptoms and others could develop new mental health problems 4,8. Even those with high levels of exposure to COVID-19 are also expected to have mental health problems 9. Different associations and health professionals around the world have expressed their concern since the beginning of this pandemic about the possibility that the increase in these mental health problems will increase suicide rates 10.

The available evidence suggests that people who are more concerned about COVID-19 and its consequences report higher levels of anxiety and depression 6,7,11, which are associated with an increased risk of suicide 10,12. Hence, suicidal ideation and its relationship with the concerns or effects derived from this pandemic are the subject of interest in this study.

According to the Pan American Health Organization (PAHO), suicide is a priority public health problem in the Americas 13. In 2019, more than 700,000 people committed suicide; that is, 1 in 100 deaths is related to this problem, which led the WHO to develop guide-lines to help prevent this phenomenon worldwide and promote care measures 14. However, this is a region in which suicide prevention requires greater government attention and awareness of the associated risks so that people can seek help.

The 2020-2025 PAHO Strategic Plan included suicide as a worrisome problem that should be addressed in the realm of public health to achieve the purposes of the United Nations' Sustainable Health Agenda for the Americas, within which the goal is to reduce premature mortality by one-third by 2030. Therefore, priority preventive actions are required to contribute to the achievement of this objective, since the COVID-19 pandemic has exacerbated the risk factors 15.

Gunnell *et al.*^10^ state that a wide-ranging inter-disciplinary response with effective approaches aimed at promoting mental health among the population is key to preventing suicides. Other authors suggest that prevention should focus on enhancing resilience and protective factors of suicide and not only on intervening risk factors 13,16.

From a multilevel and socioecological approach, it is considered that there are relational factors that have an important role in the definition of risk factors and protective factors of suicidal behavior. These are defined by the direct interaction of people, with family relationships being an essential component. In this regard, it has been found that positive communication between parents and children influences well-being and mental health 17-20. Specifically, it is known that suicidal behavior tends to decrease when family functioning improves 21.

However, although social relationships are the axes that move daily life, few studies have analyzed the influence of positive family communication in preventing suicidal behavior and the way in which dialog and the quality of family ties can be a protective factor for mental health in times of health crisis. For this reason, this article partially fills this empirical gap by presenting the results of a study focused on analyzing the mediation of open or positive family communication in the relationship between suicidal behavior and the concerns or affectations suffered in a complex and difficult period as was the first year of the COVID-19 pandemic in Colombia. This mediating approach is relevant since insufficient or scarce communication is a risk factor for suicidal behavior 22.

In this article, we focus on identifying these concerns or perceived affectations in three areas: family, economy, and health. Additionally, Olson's circumplex model of family systems were taken as a starting point to determine whether these aspects influence suicidal ideation and how the family communicative environment intervenes as a mediating variable between these problems. The starting hypotheses of this study are as follows:

H1: The concerns or affectations suffered in aspects related to health, intrafamily relations and the economy significantly influenced the high level of suicidal ideation in the first year of the COVID-19 pandemic.

H2: The effect of these concerns or affectations suffered in the pandemic on suicidal ideation is less when it is mediated by open or positive family communication.

## METHODS

This descriptive-explanatory study is based on a nonexperimental cross-sectional design. The population for this research is composed of Colombian citizens of legal age who resided in their country during the first year of the COVID-19 pandemic. The sampling method used was non-probabilistic and convenience based. Sample selection was carried out using quotas to increase the rigor of the design. The sample size was estimated based on population growth statistics for 2020. However, this type of sampling does not allow for generalization to the Colombian population. Due to the difficult public health conditions during the first year of the COVID-19 pandemic, it was not possible to carry out a probabilistic sampling. Therefore, the sample used is not random, but intentional.

Quota-type cluster sampling was carried out, with the participation of Colombians residing in the main capital cities and rural areas of the departments that are part of the three most populated regions of Colombia: Andina-Cafetera, Pacific and Caribbean.

Age ranges of 18-26 years (young people) and 28-59 years (adults) were used as quotas to define the participating sample (n=660), with a mean age of 30.92 (SD = 11.43). This sample size was calculated based on the official figures of the National Department of Statistics of Colombia, which estimated a population size of 41,052,314 in these departments in 2020, with a margin of error of 3.9% (Z=1.96; variance=0.2 5). The participant quotas of 330 for young people and 330 for adults were distributed equally, as suicide mortality rates in Colombia are significantly related to age groups and the methods used 23.

Women showed more interest in participating in this study, so they had a greater representation in the sample (62.8%) than men (37.2%). The process of collecting and processing the information complied with the ethical guidelines of the participating universities and the Ministry of Health of Colombia, which are related to the ethical principles of the Declaration of Helsinki on research with human beings. This study is endorsed by the ethics committee of the Technological University of Bolívar (Universidad Tecnológica de Bolívar) (UTB) and associated to the Research Department with code NV03PS2201.

University students who participate in research hotbeds and develop their professional careers at the UTB were trained. These students applied between November 1 and 30, 2020, the online questionnaire created for the purposes of the study to relatives, peers, friends, and acquaintances, with prior informed consent and taking into account the requirements of the sample in terms of ages and city or region of residence in Colombia.

Open family communication was evaluated using the Parent-Adolescent Communication Scale (PACS) by Barnes & Olson 24, which contains six items that inquire from 1 (never) to 4 (very frequently) the presence of family openness indicators such as freedom of expression, credibility, attention, talking about problems and expressions of affection. These indicators explain 63.1% of the total variance of this type of positive communication. The instrument has factorial validity (KMO=0.882; Chi2 = 19 442,6; df=15; p=0.000) and a good level of reliability (Cronbach's alpha =0.882) with the data collected.

With the Positive and Negative Suicide Ideation Inventory (PANSI) by Osman *et al.*^25^, this problem was evaluated through eight items that measure, in a range of 1 (never) to 5 (always), frequent negative thoughts associated with the desire to kill oneself because of not meeting the expectations of others, hopelessness, crisis in relationships, inability to solve problems, feelings of failure and despair. These items explained 76.35% of the total variance in suicidal ideation. The scale has good reliability indices (Cronbach's alpha=0.953) and factorial validity (KMO=0.938; Chi^2^=536.5; gl = 28; p=0.000).

The concerns or perceived affectations in this pandemic were evaluated using a dichotomous response format, using the EPEP (for its acronym in Spanish) scale by Garcés-Prettel *et al.*^26^, which contains nine items that explain 54.9% of the total variance of concerns felt at the family, economic and health levels. This scale has values of reliability (KR20=0.639) and factorial validity (KMO=0.662; Chi^2^=764.5; df=36; p=0.000) that are acceptable for exploratory studies of this nature that explore emerging issues associated with the COVID-19 pandemic.

To confirm the first hypothesis, a percentile scale was developed for the data obtained from the PANSI scale, which allowed us to establish three levels of suicidal ideation (low, moderate, and high) in the subjects participating in this study, taking as reference the minimum and maximum values of the general score and the 30th and 70th percentiles. Subsequently, the high level of ideation was defined as a dependent variable, assigning the value i and 0 to the rest of the levels. The concerns or affectations suffered in the first year of the pandemic were included as independent variables in a binary logistic regression model.

To confirm the second hypothesis, a simple mediation analysis was performed using model number four proposed by Hayes 27, which works using the Process Macro, which was installed in SPSS version 25. The direct effect size obtained from the relationship between the concerns or affectations suffered and high suicidal ideation was reviewed. It was then compared with the size of the indirect effect mediated by open family communication. The differences were analyzed through the values of the lower (BootLLCI) and upper (BootULCI) limits of the confidence interval.

## RESULTS

### Levels of suicidal ideation

The results obtained reveal that 35.2% of the Colombian participants had a high level of suicidal ideation in the first year of the COVID-19 pandemic. A total of 40.8% presented a moderate level and 24.1% a low level. When relating these levels to the age ranges established as a quota within the sample, it was found that young people between 18 and 26 years had a higher percentage of high ideation (25.1%) than adults between 28 and 59 years (10%).

The risk estimate showed an advantage ratio of 4.04 (CI=between 2.86 and 5.71). This means that the probability of having high suicidal ideation in this evaluated pandemic context was four times higher in young people than in adults. Likewise, participating women (23.8%) showed a higher percentage of high suicidal ideation than men (11.1%), with an advantage ratio of 1.44 (CI=be-tween 1.03 and 2.02). In both cases, the Mann-Whitney U test showed that these differences were statistically significant with a p-value (p)=0.000.

### Relationship between suicidal ideation and concerns or affectations suffered in the pandemic

The data shown in Table 1 reveal that in the realm of health, the greatest concerns or affectations suffered in the first year of the pandemic had to do with the risk of infection by COVID-19. In the realm of intrafamily relationships, problems of coexistence in the home and the negative effects of distancing and quarantine on social and family ties were the main reasons for concern. At the economic level, the most frequent concern or affectation was related to the financial crises suffered.

**Table 1:** Concerns or perceived affectations predictive of high suicidal ideation

Factors and indicators	Yes (%)	No (%)	B	EN	Wald	gl	Exp
Health realm							
I have been concerned about my relatives getting infected	89.8	10.2	-0.23	0.34	0.44	1	0.79
I have been concerned about the risk of personal infection	78.5	21.5	-0.47	0.26	3.1	1	0.62
I have had health problems	39.2	60.8	0.79***	0.19	16.9	1	2.2
Intrafamily relations realm							
The distancing and quarantine affected me at the family level	45.5	54.5	0.44*	0.19	5.0	1	1.5
I have experienced a crisis due to family coexistence problems	40.3	59.7	0.42*	0.20	4.2	1	1.5
I have had family losses (deaths)	26.1	73.9	0.35	0.20	2.8	1	1.4
I have suffered due to separations or family breakdowns	19.5	80.5	0.59*	0.23	6.1	1	1.0
Economic realm							
I have suffered financial crises or concerns	60.5	39.5	0.04	0.21	0.05	1	1.0
I have been concerned because I do not have a job	45.6	54.4	-0.19	0.21	0.83	1	0.82

Source: Self-elaboration; e Binary logistic regression coefficients: ^*^p<0.05; ^**^p<0.01; ^***^p<0.001.

By relating these concerns or perceived affectations in the first year of the COVID-19 pandemic with suicidal ideation, the first hypothesis was accepted based on the values obtained in the binary logistic regression coefficients (B) and in the values obtained in the omnibus test of the coefficients of the model with all the indicators evaluated in each area (Chi^2^=137.8; gl=10; p=0.000) (Table 1).

However, the significant predictors that most strongly explain the high suicidal ideation in this evaluated pandemic context come from the realm of health and intrafamily relationships, and influence between 12.8% (R^2^ Cox and Snell) and 17.7% (R^2^ Nagelkerke) in the total variance. The predictive capacity of this model increases to 25.9% when including the age of the subjects (B=1.33; p=0.000), which was the sample quota and at the same time was shown to be a variable that increases the risk of suicidal ideation in the case of young people.

However, when specifically analyzing the behavior of the model, we found that concern for health problems is the significant predictor that most influences high suicidal ideation (B=0.793; p=0.000). They are followed in their order at the intrafamily level by concerns about the separations or breakdowns suffered as in the case of divorces (B=0.593; p=0.013), the effects on family relationships generated by distancing and quarantine (B=0.440; p=0.024) and problems with family life (B=0.422; p=0.040), which also increased the high levels of suicidal ideation.

### Open family communication as a mediating factor

In this pandemic context, the participants evaluated family communication positively. When the frequent and very frequent options are added, it was possible to establish the presence of family openness indicators such as freedom of expression (70.4%), credibility (80.1%), attention (82.5%), talking about problems (73.5%) and expressions of affection (72.5%).

The values presented below were obtained taking as a reference the significant predictors of the previous logistic regression model. Open family communication was the mediating variable used to study the relationship between significant concerns or affectations and high suicidal ideation.

The data presented in Table 2 confirm the acceptance of the second hypothesis, since the effect of the concerns or perceived affectations in the pandemic at the family and health level on suicidal ideation showed a significant decrease when there was open or positive family communication in the homes. This can be seen when comparing the size of the direct effect with the indirect effect that was statistically significant in the four mediation models evaluated, where the lower (BootLLCI) and upper (BootULCI) limits of the confidence interval do not include the zero value, as suggested by the literature 27.

**Table 2:** Concerns or perceived affectations predictive of high suicidal ideation and mediating effect of family communication

Mediation models with analysis variables	Realm	Boot LLCI	Boot ULCI	Direct effect	Indirect effect
Mediation Model 1: Direct influence of the concerns or perceived affectations in the pandemic by health problems on suicidal ideation and indirect effect of open family communication.	Health	0.17	0.47	0.94	0.30
Mediation Model 2: Direct influence of concerns or perceived affectations in the pandemic by family separations or breakdowns on suicidal ideation and indirect mediating effect of open family communication.		0.02	0.19	0.74	0.35
Mediation Model 3: Direct influence of concerns or perceived affectations in the pandemic by distancing and quarantine on suicidal ideation and indirect mediating effect of open family communication.	Intrafamily relationships	0.07	0.32	0.66	0.19
Mediation Model 4: Direct influence of concerns or perceived affectations in the pandemic by problems of family coexistence on suicidal ideation and indirect mediating effect of open family communication.		0.26	0.57	0.58	0.41

Source: Self-elaboration.

In the case of mediation model number 1, it can be seen by dividing the size of the indirect effect with the direct effect that the impact of health concerns or affectations on suicidal ideation decreases by up to 32.4% when family communication is open. In Models 2, 3 and 4 that have to do with the effect of the concerns or affectations perceived at the level of intrafamilial relationships on suicidal ideation can decrease between 29.1% and 70.1% when communication is open or positive among household members.

## DISCUSSION

The results of this study contribute to the literature on mental health, a new perspective focused on analyzing the influence of concerns and felt affectations associated with COVID-19 on suicidal behavior and the mediating role of open or positive family communication as a protecting factor.

Suicidal ideation is a risk factor for suicidal behavior, and it is identifiable and modifiable; hence, it is important in preventing this problem 28. It has been expected that due to isolation, quarantine, unemployment, and stress due to the restrictive measures implemented during the COVID-19 pandemic, there will be increases in suicidal ideation and behavior among at-risk populations 29.

Our study reported elevated levels of suicidal ideation during the first year of the COVID-19 pandemic in participating Colombians. These levels are above the prevalence rates reported by countries such as Greece, Spain, United States, Norway, Thailand, Indonesia and Taiwan, China and Bangladesh ranging between 5.2% and 26.7%.

The young participants in our study are the population at greatest risk, which is consistent with other previous studies that show a higher level of suicidal ideation in early adulthood, especially young people between 18 and 26 years of age 30,31. These findings are a warning sign for the entities responsible for ensuring mental health.

Faced with this context of health crisis, the literature indicates that people most concerned about COVID-19 and its consequences experience greater affectations on their mental health 6,7,11,12. In fact, in our case, it was found that in the realm of health, concerns about general health problems predicted high suicidal ideation with greater force. Likewise, the risk of infection by COVID-19 was one of the most frequent concerns, which coincides with other studies that have also considered it a risk factor for suicide 10.

On the other hand, in the economic sphere, the evidence shows that concerns about the economic consequences of the pandemic are the strongest predictor of mental health deterioration during the first stage of the pandemic 7. However, most of this evidence comes from countries with conditions of well-being superior to those of Colombia and reveals how unemployment and the economic consequences derived from the pandemic are risk factors for suicide 32. In our case, economic concerns (including unemployment), although they had a high percentage of frequency, did not predict high suicidal ideation. This finding may be related to the fact that people adapt to crisis situations over time 33 and more in countries such as Colombia, where 39.3% of the population suffers from monetary poverty and an informal occupation greater than 40% according to national government statistics reported by the National Administrative Department of Statistics (Departamento Administrativo Nacional de Estadística) (DANE) in 2022.

Regarding the family environment, the most frequent concerns of the participants had to do with the family affectation felt by the problems of family coexistence and the measures of social distancing. These concerns, including family separations and breakdowns, were shown to be predictors that significantly increase suicidal ideation. However, we found that open family communication can reduce the negative effect on suicidal ideation, health concerns and family problems related to distance, breakdowns, and coexistence.

Faced with the aforementioned family findings, there is evidence that reaffirms how social support is beneficial for health 34. On the other hand, it is known when the family environment is mediated by hostile conflicts, this influences the development of suicidal behavior 21. Likewise, Dávila-Cervantes & Luna-Contreras 35 confirm that when the family climate is unfavorable for adolescents, there is a greater propensity to attempt suicide that worsens even more when there are low levels of positive communication between parents and children.

In conclusion, our findings highlight the protective function of good communication between household members to contribute to the prevention of suicide and to better manage concerns or affectations suffered in this health crisis. It is worthwhile to further strengthen mental health prevention programs during and after a pandemic, giving greater importance to the promotion of good treatment and assertive, affective, and empathetic family dialog to achieve greater attention and openness that allows speaking openly about the problems.

This type of open or positive communication could help to stimulate the processes within the family and the effective solution of the differences and problems that arise in this context of health crisis. This is especially because the evidence indicates that low trust in communication with parents is a factor associated with suicidal behavior in adolescents 22, which was the population at greatest risk not only in our study but also at the global level according to the data of the WHO in 2016. Dialog and collective coping strategies can be effective resources to help reduce stress in the family and better manage situations or concerns in this time of the pandemic surrounded by fears and uncertainties 26 ♣
